# Person-centered clinical method: application in the occupational context

**DOI:** 10.47626/1679-4435-2024-1340

**Published:** 2025-08-25

**Authors:** Dimitrios Nikolaos Georgopoulos Filho, Edvard Izidro dos Anjos Junior, Rubens Jonatha dos Santos Ferreira, Julia Lujan Pichamoni, Fabio Dezo, Daiane Aparecida Dias

**Affiliations:** 1 Population Health, Hospital Sírio-Libanês, São Paulo, SP, Brazil

**Keywords:** occupational health, health promotion, patient-centered care, occupational medicine., saúde ocupacional, promoção da saúde, assistência centrada no paciente, medicina do trabalho.

## Abstract

Health promotion, which is important for health policies in Brazil and worldwide, enhances patient self-awareness and autonomy, strengthening the relationship between health care professionals and patients by negotiating care priorities and goals, resulting in the efficient use of health resources. These principles align with the person-centered clinical method, a theoretical framework that offers techniques to assist health care professionals in clinical practice, especially physicians. This method positions the patients as the protagonists of their own health, actively participating in setting priorities and making decisions. However, when applied by occupational physicians, the person-centered clinical method still involves gaps and challenges to integrating prevention and health promotion, although it can add value to occupational health examinations, promote health within companies, and ensure the overall functionality of workers and the rational and sustainable use of health services. Patient-centered examinations not only meet Regulatory Standard 7 requirements but also provide an efficient examination and comprehensive care for workers that extend beyond work activities. The person-centered clinical method fulfills legal mandates and enhances the overall health of workers, fostering a healthier and more productive work environment.

## Abstract (pt)

No cenário das políticas de saúde no Brasil e no mundo, a promoção da saúde desempenha um papel crucial, fundamentando o planejamento e a execução de ações nessa área. Entre os pilares dessa prática, destacam-se a elevação da autoconsciência e da autonomia dos indivíduos, o fortalecimento do vínculo entre profissional e paciente, com negociação de prioridades e metas de cuidado, e o uso eficiente dos recursos de saúde. Esses princípios estão alinhados ao método clínico centrado na pessoa, um arcabouço teórico que oferece técnicas para auxiliar profissionais de saúde, especialmente médicos, na prática clínica. Esse método coloca o paciente como protagonista de sua saúde, destacando sua participação ativa na definição de prioridades e na tomada de decisões. No entanto, ainda há um déficit na aplicação do método clínico centrado na pessoa pelos médicos do trabalho, bem como desafios na integração da prevenção e da promoção da saúde no contexto ocupacional. Este estudo visa relatar a implementação desse método nos atendimentos ocupacionais, com o objetivo de agregar valor aos exames ocupacionais, promover a saúde dentro das empresas, assegurar a funcionalidade global dos colaboradores e utilizar os serviços de saúde de forma racional e sustentável. Um exame ocupacional centrado no paciente vai além do cumprimento das exigências legais da Norma Regulamentadora 7, proporcionando uma avaliação ocupacional eficiente e um cuidado integral ao colaborador, transcendendo sua atividade laboral. Essa abordagem não apenas cumpre os requisitos legais, mas também valoriza a saúde integral do trabalhador, promovendo um ambiente de trabalho mais saudável e produtivo.

## INTRODUCTION

Regulation and government policy are important drivers of change in current practice. European Standard (Slovenian Institute for Standardization) 17398:2020, “Patient Involvement in Health Care - Minimum Requirements for Person-Centered Care”, was developed by the European Committee for Standardization/Technical Committee, building on the core dimensions of person-centered care in the University of Gothenburg model. This standard aims to facilitate the introduction, development and research of this approach by different actors in health services, patient organizations, researchers and companies.^1^

This standard can guide managers and administrators, as well as actors in the political system, regarding goals and programs with the dimensions necessary for person-centered practice. Therefore, it can be considered a support tool in bottom-up or top-down activities.^1^

The Brazilian National Policy on Worker Health (regulated by Ordinance 1,823, August 2012) establishes principles and guidelines for worker care, including comprehensiveness, longitudinality, and community participation. This ordinance also establishes a link between individual actions and care interventions among the determining factors of worker health, reinforcing a view that is both collective and individualized within a broader context that considers individuals beyond their work experience.^2^

The specificities of the work-based health-disease relationship require an approach that goes beyond a multicausal explanatory model, understanding the pathological process as a more complex relationship. From this perspective, potential dangers must be considered in relation to historicity, psychological burdens, subjectivity and other sociopsychological, biological, and spiritual factors.^3^

The historical process of changing the paradigm of occupational medicine and occupational health to worker health in Brazil has definitive milestones that identify the health-disease process as a structural phenomenon emerging from working conditions. Thus, the biomedical model, whose nature is authoritarian and hierarchical, is theoretically limited in its analysis of worker health.^4^

In this scenario, a new dimension of health care for the working class has been expressed, with workers taking the lead in applying prevention standards and promoting the investigation, development, and implementation of the measures necessary for health surveillance and the preservation of their physical integrity.^3,4^ Worker health is the only perspective that, by freeing itself, also emancipates the health of other individuals. Although this idea was propounded in Brazil in the 1970s, a model subservient to disease-centered technical hierarchy and information systems still persists.^4^

To ensure a comprehensive approach to the individual, a person-centered care model is currently being sought in the health care context. Humanization in health care has been discussed since the 1990s as an important tool for changing the scenario of doctor-centered care, allowing patients to be the protagonists of their care. Thus, care models are now based on the individual, rather than the professional.^3^ In contrast to an exclusive focus on the organic components of the disease, which is characteristic of a paternalistic care model where the health professional occupies the center of care, a concern with holistic care emerges.^5^

The person-centered clinical method proposes constructing care processes from the perspective of patients, while health care professionals support their care journey. This method, in addition to strengthening patient engagement, also shifts the focus of care away from the physician and promotes greater co-accountability. This approach is based on the understanding that patients should be at the center of care and, by actively participating in health care, they stop being passive recipients and assume an active role in managing their health and self-care.^5,6^

Thus, patient engagement is a crucial health care resource due to its benefits for clinical outcomes and the sustainability of health care.^7,8^ Engaged individuals tend to be committed to understanding their health condition and their role in the treatment outcomes. Since better treatment outcomes lead to improved recovery of work capacity, patient engagement is an important goal of clinical care, health education, and care planning.^7^ Studies indicate that, without engagement, professional-patient communication is impaired and treatment adherence and outcomes worsen, as does care quality.^7,8^

For greater patient participation in their own care, the culture of health services must change, so that professionals can encourage and support a more active attitude among patients, considering them as partners capable of playing a responsible role in their health and care.^3^ Health care engagement is recognized worldwide as a key strategy for improving treatment adherence, clinical outcomes, and satisfaction with care.^6,9,10^ Well-informed patients who are directly responsible for managing their care are essential to making health care organizations more sustainable on economic, organizational, and psychological levels.^10^

Major global reference centers, health providers, operators, health specialists, managers, and policy makers are recognizing the importance of changing the planning and delivery of health care to allow patients a more active role in managing their care.^6,10,11^ This active role in health self-management is becoming increasingly important for safe and quality care. Measuring and promoting patient involvement is now a priority for health systems around the world.^5^ However, studies show that there is a gap in scientific research regarding the implementation and assessment of initiatives to increase patient involvement in health care.^11-13^

## METHODS

The person-centered clinical method, developed by Stewart et al.^14^ includes four components: exploring health, illness and the experience of illness; understanding the person as a whole; developing a joint plan to manage problems; and strengthening the relationship between the person and the occupational health professional. Chart 1 further describes the four steps.

**Chart 1:** The four interactive components of the person-centered clinical method

Exploring health, illness and the experience of illness
Personal and unique perceptions and experiences of health (meaning and aspirations) Clinical and work history General physical examination, focused on complaints and work activity Complementary examinations, including those necessary for work activity and recommended in the Occupational Health Medical Control Program (when applicable) Dimensions of the experience of the disease (feelings, ideas, expectations and overall functionality, including the worker’s activity)
Understanding the person as a whole
The person (life story and personal and developmental issues) The immediate context (family, work, exposure to occupational hazards, and social support) The broader context (culture, community, and ecosystem)
Developing a joint plan to manage problems
Problems and priorities Treatment and/or management goals The roles of the person and the professional Assessment of suitability for work in relation to needs such as (re)adaptation, work leaves, and ergonomic guidelines

Compassion and empathy Power Healing and hope Self-knowledge and practical wisdom Transference and countertransference

Source: Adapted from Stewart et al.^14^

Although the person-centered clinical method considers the workers’ occupational context within their immediate socioeconomic context, this must be actively explored through questions about adaptation to the work regime, the work activities, and any difficulties or incapacities in performing these activities.

The person-centered clinical method is a tool for establishing more effective communication with the patient, forming bonds and shared responsibility. However, in the occupational context, it is necessary to perform physical examinations that focus on subclinical conditions that may be aggravated by work activities or present a risk of illness due to work organization, even when no specific complaints have been made.

### CARE COORDINATION IN OCCUPATIONAL EXAMINATION

Care coordination promotes improved care quality, increasing access to different levels of care and integrating actions and regional health services.^15^ The conceptual model of McDonald et al.^15^ accurately illustrates the meaning attributed to care coordination: “anything that bridges gaps”. Thus, coordination means establishing connections to achieve the higher objective of meeting patient needs and preferences in care provision with value and quality. The act of coordinating implies the deliberate organization of activities that involve two or more people (including the beneficiary of the service/health system) and the management of resources to produce adequate care.

Care coordination is at the heart of this process, linking community, health care, pharmaceutical, and other resources, keeping the person at the center and supporting the implementation of the agreed-upon care plan. In this scenario, the higher the number of people and services involved in care provision, the more complex interventions to resolve a given problem become, and the higher the level of coordination required to achieve the desired result.^15^ For example, chronic conditions require the simultaneous use of several services. Thus, coordination cannot be seen as static or as a given. On the contrary, to be effective, it must be conceived from a dynamic perspective, adjusted to the system’s specificities, complexity, and fragmentation level and, we would add, to the particular and singular characteristics of the groups and individuals for whom the system exists. Hence, coordination presupposes the construction of dynamic networks, requiring cooperation and integration of the involved actors and services.

Coordinating care is challenging on all levels, especially in occupational services, where the patient is often faced with a fragmented network and disconnected services, as opposed to a journey followed exclusively in the public health system. Another challenging point is to broaden the perspective of workers to allow assessments that go beyond occupational diseases and risks, adopting a comprehensive and holistic approach that considers patient needs and care preferences.^15^

The integration of worker health care with other regional resources is part of the territorialization concept of the Brazilian model, which is also described in the National Policy on Worker Health.^1^ In fact, a model in which occupational health teams work in partnership with primary health care teams can be considered a change in health practices toward new objectives.^16^ In view of this, it is necessary to understand occupational health as a multidisciplinary outpatient clinic that integrates occupational examinations and the precepts of primary health care. In addition, it is essential to incorporate the regional population as an object of intervention, understanding it as a dynamic and living space for establishing social relations. Thus, the workers’ immediate environment is to be recognized as their territory.

In community care, it is important to recognize the factors that influence the disease processes specific to that population, such as the workplace, exposure to risk factors, and workload. These processes must be adapted to each person and can be carried out through mapping and assessing geographic and environmental conditions related to work and health potential to organize individual and/or collective actions, depending on the observed characteristics and problems.^16,17^

As recommended by the National Network for Comprehensive Care for Worker Health, community resources, such as the supplementary health network, the Unified Health System, and health services offered by the company and its occupational outpatient clinic, can be coordinated. When these different systems and services act jointly, comprehensive health care and the rational use of services are guaranteed. This process is challenging, since each part of the system has different objectives.^1,4^

These different perspectives must be aligned to strengthen the care network and, hence, comprehensive care, avoiding fragmentation of the system. We highlight the need for better integration of occupational medicine into this network since more than mandatory notifications or interventionist medicine will be needed to strengthen the care network. In the occupational context, this process should be the first step towards building person-centered comprehensive care. The current socioeconomic relevance of disease prevention and health promotion at the primary care level has been discussed by the World Health Organization^17^:

“Mainstream health care systems around the world are failing to keep up with the trend of declining acute conditions and rising chronic conditions. When health problems are chronic, the acute care model does not work. The acute care paradigm currently dominates among decision-makers, health care workers, administrators and patients. To address the rise of chronic conditions, it is imperative that health care systems move beyond this dominant model.”^17^

Furthermore, as pointed out by Ribeiro & Cavalcanti,^18^ care coordination prevents duplication of services and unnecessary procedures. When different health services are well coordinated, there is less likelihood of redundant tests or incompatible medications, which not only contributes to sustainable resource use, but also reduces potential risks to the patient.

Therefore, we believe that care coordination is a critical attribute for optimizing health services. It ensures that people receive care in an integrated manner, effectively focusing on their needs, avoiding gaps in treatment, improving care continuity, and reducing wasted resources. Stimulating such a culture in occupational health will be an important step in building person-centered occupational care, no matter how long and complex the journey may be. Revisioning occupational health in light of comprehensive care can be a driving force for systemic change and can be a key point in improving health care for workers, directing them to coordinated care and acting as a gatekeeper for primary care services within the scope of public or supplementary health, in addition to ensuring timely access to these services.

### MOTIVATIONAL INTERVIEWING AS A TOOL FOR SHARED CARE

Motivational interviewing is an evidence-based approach that encourages patients to change their lifestyle and commit to behavioral change. In this approach, the professional relates to the patient in an empathetic and non-confrontational manner through a collaborative bond that leads to dialogue, respecting the patient’s choices and resolving ambivalence about the changes needed to improve a given health condition.^19^ Motivational interviewing can be adapted to the individual, the problem, and the culture, and can be conducted by trained professionals in different scenarios. Thus, in the health care context it can be used to propose new behaviors and change habits that encounter resistance from a considerable segment of the population and directly impact health and quality of life.

Therefore, it is essential to develop alternative strategies to increase patient motivation to take care of their own health and that can be used in different situations.^19,20^ In view of this, it is important to highlight the important role of health care professionals in guiding and educating patients about behaviors associated with self-care and health promotion. However, understanding and contributing to adherence is a complex process that includes not only monitoring and compliance with the prescribed treatment, but also the co-responsibility of patients in defining their care plan and lifestyle changes. Thus, self-care and improving treatment adherence are processes that require, first and foremost, the patient’s intrinsic motivation, and motivational interviewing can be used to this end.^20^

## CONCLUSIONS

Occupational health teams can play a decisive role, not only in aspects related to the worker capacity, but in disease prevention and health promotion for this population, acting as an important segment in collective health. Considering complexity of agents and service providers who act in the lives of workers, we propose, as an initial step, using the person-centered clinical method and motivational interviewing as tools to enable care coordination and a comprehensive view of the person, to adapt the specific processes and attributes of occupational medicine and allow for a holistic view of care delivery. Effective care coordination not only benefits individual workers, but can also lead to significant improvement in productivity and general well-being, positively affecting not only physical health but mental health, quality of life, and work engagement, as well.

Making occupational examinations mandatory can guide the attention of occupational physicians and enable preventive health actions, leading to the correct use of health resources, patient engagement in self-care, and, consequently, in overall functionality. This will not only result in better health care, but it will contribute to the sustainability of services, whether public or private. Using the person-centered clinical method and motivational interviewing in occupational medicine will benefit clinical practice and improve the organization of networked care by placing the patient at the center. This makes it possible for employers to reduce indicators of absenteeism in both the short and long term, in addition to strengthening trust in the company’s health services.
